# Structure and Composition of the Cuticle of the Goose Barnacle *Pollicipes pollicipes*: A Flexible Composite Biomaterial

**DOI:** 10.3390/md21020096

**Published:** 2023-01-29

**Authors:** Mariana Almeida, Emanuel M. Fernandes, Catarina F. Marques, Flávia C. M. Lobo, Rita O. Sousa, Rui L. Reis, Tiago H. Silva

**Affiliations:** 1 3B’s Research Group, I3Bs–Research Institute on Biomaterials, Biodegradables and Biomimetics, University of Minho, Headquarters of the European Institute of Excellence on Tissue Engineering and Regenerative Medicine, AvePark, Parque de Ciência e Tecnologia, Zona Industrial da Gandra, Barco, 4805-017 Guimarães, Portugal; 2 ICVS/3B’s–PT Government Associate Laboratory, 4806-909 Braga/Guimarães, Portugal

**Keywords:** Cirripedia crustaceans, chitin, marine polymers, integument, exoskeleton, biomimetic model

## Abstract

Arthropods, the largest animal phylum, including insects, spiders and crustaceans, are characterized by their bodies being covered primarily in chitin. Besides being a source of this biopolymer, crustaceans have also attracted attention from biotechnology given their cuticles’ remarkable and diverse mechanical properties. The goose barnacle, *Pollicipes pollicipes*, is a sessile crustacean characterized by their body parts covered with calcified plates and a peduncle attached to a substrate covered with a cuticle. In this work, the composition and structure of these plates and cuticle were characterized. The morphology of the tergum plate revealed a compact homogeneous structure of calcium carbonate, a typical composition among marine invertebrate hard structures. The cuticle consisted of an outer zone covered with scales and an inner homogenous zone, predominantly organic, composed of successive layers parallel to the surface. The scales are similar to the tergum plate and are arranged in parallel and oriented semi-vertically. Structural and biochemical characterization confirmed a bulk composition of ɑ-chitin and suggested the presence of elastin-based proteins and collagen. The mechanical properties of the cuticle showed that the stiffness values are within the range of values described in elastomers and soft crustacean cuticles resulting from molting. The removal of calcified components exposed round holes, detailed the structure of the lamina, and changed the protein properties, increasing the rigidity of the material. This flexible cuticle, predominantly inorganic, can provide bioinspiration for developing biocompatible and mechanically suitable biomaterials for diverse applications, including in tissue engineering approaches.

## 1. Introduction

Chitin is an abundant renewable polymer in the marine environment, and it may be found in a variety of marine groups/taxa, as in the skeletons of some demosponges [1,2] and black corals [3,4], in the beaks of cephalopods [5] and widely across crustacean cuticles [6]. In crustaceans, these cuticles are formed by the integument, which comprises the epidermis and its productions, and usually forms an exoskeleton, consisting of an organic matrix of chitin, pigments, structural and acidic proteins and an inorganic fraction predominately of calcium carbonate [7,8]. Nonetheless, there are variations in this basic pattern due to the different functions cuticles may have related to the animal body plan, habitat and behavior [9]. For example, lobsters with a fast swimming escape response require less mineralized exoskeletons than crabs that burrow into sediments to escape [8]. These differences are manifested across major crustacean taxa and even among different species of particular taxa or within individuals, depending upon the mechanical and physiological function of the cuticle in different regions of the body [10,11]. For instance, the cuticle of lobsters is heavily mineralized in the carapace and is thin, pliable and almost non-mineralized between the segmented rigid parts, greatly contributing to their mobility [8,12]. In another example, the appendages specialized for striking prey such as the ones used by aggressive crustacean stomatopods (commonly called “mantis shrimps”) require tailored properties such as extreme hardness or fracture toughness that are adapted to the kind of prey a species feeds on [13]. These appendages are also composed of stiff yet flexible cuticle elements to provide high elastic energy storage [14].

In the biomedical field, chitin has been explored given its biodegradability, biocompatibility, non-toxicity and improved antimicrobial activity [15,16]. Crustacean-derived chitin characterization is best known for crab and shrimp cuticles forming exoskeletons, resulting from the food processing sector, and it is used usually in the form of powders for the preparation of chitosan-based biomaterials for a variety of tissue engineering and regenerative medicine approaches [17]. Other processing forms of chitin, such as the production of pre-designed scaffolds found in skeletons of marine sponges [18] and “ready-to-use” chitin membranes from “soft-shell” crustacean exoskeletons resulting from molting [19], have been recently investigated. Interestingly, crustacean exoskeletons, which form a continuum from heavily mineralized to non-mineralized elements, have also been investigated as model biological structures for bioinspired materials engineering due to their diverse mechanical properties [10].

Crustaceans are a large and diverse group inhabiting all marine environments. Some taxa exhibit a variety of body shapes and adaptations to particular habitats and environmental conditions that are distinct from the general morphology, which is usually characterized by segmented bodies covered by an exoskeleton. Therefore, it is very interesting to understand crustaceans’ functional morphology and tailored properties to identify novel structures aiming to provide knowledge for bioinspired materials’ design by integrating functional biological structures with biomimetics.

**Figure 1:**
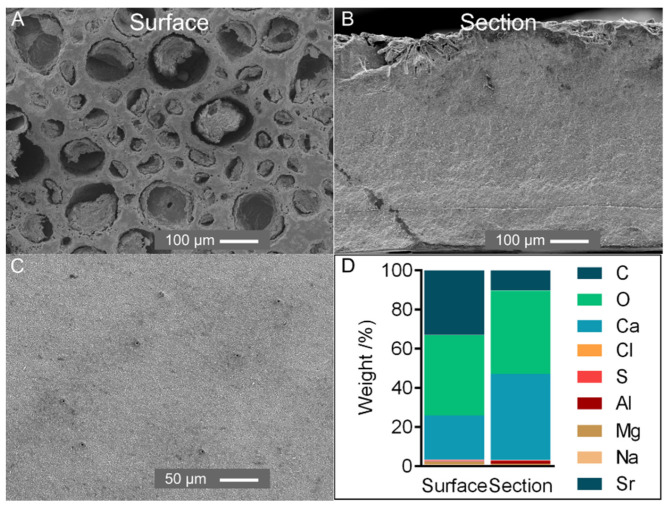
(**A**,**B**) SEM analysis of the external surface (**A**) and section of the tergum plate (**B**) (magnification ×150); (**C**) SEM analysis of the external surface of a recently formed tergum plate (magnification ×300). Surface: external surface of the plate; section: cross-section exposing the internal part. (**D**) Elemental composition weight (%) of the tergum plate of *P. pollicipes*.

Barnacles (Infraclass Cirripedia) lack the appearance of typical crustaceans as they adopt a sessile lifestyle as adults and most forms secrete “shell-like” covering plates, a trait that led some earlier zoologists to consider barnacles as mollusks. The ability of barnacles to produce mineralized structures and to adhere to wet surfaces has led to research on these animals given the interest in their cement proteins and also to better understand the interaction of minerals and organic components, relevant for the design of new biomimetic materials [20,21,22]. On the other hand, some barnacles are local economic resources, such as the goose barnacle *Pollicipes pollicipes*, a highly prized seafood product collected on rocky shores of Spain and Portugal. This filter-feeding animal has a restricted range distribution, from the Atlantic coast of France to North Africa, forming clusters of sessile individuals in exposed intertidal and subtidal zones to benefit from food transport while supporting the challenging mechanical settings of high-energy waves and the pressures of periodic desiccation [23]. They are characterized by a flexible muscular peduncle attached to the substratum at one end and carrying the capitulum, which encloses the soft parts with plates, at the other end [24]. Early reports described the structure of the integument of the genus *Pollicipes* that confers protection to the body. It is divided into two regions, one covering the surface of the capitulum which incorporates several plates, and one covering the peduncle (Figure 1). The latter has been described as a tick and laminated chitinous cuticular layer containing calcified scales [25,26]. To the best of our knowledge, detailed structural elements of this composite are not known.

This work aims to characterize the plates and cuticle of the crustacean *P. pollicipes* and contribute to the understanding of crustacean functional biology and primarily provide knowledge to the field of marine origin biomaterials and biomimetics. Firstly, the composition and organization of the tergum plate and cuticle were analyzed by imaging techniques (histology and/or SEM) and determined their chemical components. After that, untreated and treated cuticles—subjected to decalcification and deproteinization—were subjected to a combination of X-ray diffraction, FTIR spectroscopy, thermogravimetric analysis, SEM and mechanical testing for a fine structural characterization.

## 2. Results and Discussion

### 2.1. Characterization of Tergum Plate

Elemental composition was assessed on the *P. pollicipes* tergum plate. Most of the plate comprised Ca, O and C, with all other elements occurring at less than 1.5 wt% (Figure 1A). In a cross-section, calcium was the main element, whereas the surface showed a high relative composition of carbon, possibly related to microbial/diatom colonization as observed in the cuticle of the stalk (Supplementary Material, Figure S1).

An X-ray revealed one sharp peak at 29° and other small peaks at 36°, 39°, 43°, 47° and 48° assigned to calcite, ICDD card numbers #01-085-1108 [27] (Supplementary Material, Figure S2). This result follows other crustacean and marine invertebrate calcified structures, which generally adopt calcium carbonate for skeleton reinforcement [8,28]. SEM micrographs revealed a compact homogeneous structure composed of grains with a few μm in size (Figure 1B) and a surface with porosities (<100 µm) possibly resulting from erosion. However, in a recently formed plate, the small and regularly placed pores observed could transport material for scale development (Figure 1C).

The above results indicate that the *P. pollicipes* plate composition and structure were similar to other barnacle plates [29,30] and did not reveal a feature of interest for biomedical applications (e.g., internal porosity). Therefore, this study continued with a detailed analysis of the cuticle.

### 2.2. Composition of the Cuticle

The crustacean cuticle consists of three major components—chitin, proteins and calcium carbonate—and the relative composition of their organic and inorganic constituents and structural organization varies according to the taxa/species and within a species with the body parts [8,9,11].

Chitin has been extensively characterized on decapods, namely crabs, shrimps and lobsters, with early works reporting its presence in the cuticle of *P. pollicipes* [31]. The isolation of chitin from crustacean by-products (seafood processing industry) usually involves demineralization and deproteinization steps [32]. The former is an acid treatment that removes minerals associated with the structure by promoting the solubilization of calcium carbonates, whereas the latter is an alkaline treatment that removes the proteins bound together with all constituents by promoting their hydrolysis and further dissolution. In addition, a decolorization step may be applied to remove residual pigments. The parameters for acid and alkaline treatments (e.g., chemical, concentration, time) depend on the chitin source. For instance, as the cuticle of shrimps is thinner, the treatments for chitin extraction are moderate compared to crabs’ chitin isolation requirements [32]. The treatments carried out in this work followed the traditional ones for chitin isolation; nevertheless, optimization procedures were not considered as the purification of chitin was not the focus of this study.

The cuticle of *P. pollicipes* presented a higher content of minerals followed by proteins, carbohydrates and lipids, suggesting that the mineral scales covering the organic part represent most of the cuticle components. The proximate composition of the cuticle (Table 1) and chitin content, 9.9%, is in the range of values described for decapods despite the large morphological differences between this group and barnacles. In the rigid skeletons of commercial crab species, ash was also the principal constituent and accounted for approx. 70 %, followed by protein counting approx. 13 to 20% [33]. Chitin is also in the range of values found in commercial crabs and shrimps (9.7–20.7%), as well as seen in small shrimp-like crustacean groups (e.g., amphipods 11–12%) and insects’ cuticles (10–20%) [34,35].

**Figure 2:**
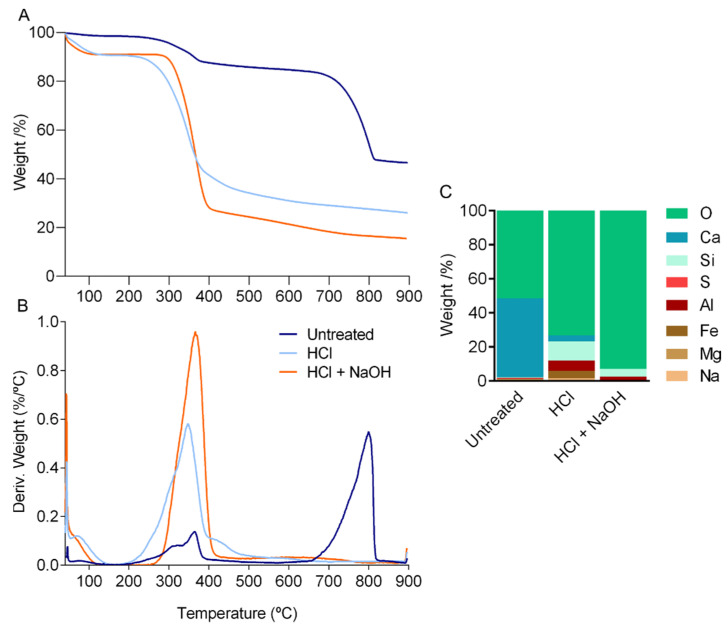
Thermal analysis of the cuticle of *P. pollicipes,* (**A**) thermogravimetric (TGA) curves and (**B**) differential thermogravimetric (DTG) curves. (**C**) Elemental composition weight (%) of the cuticle of *P. pollicipes* after thermogravimetric analysis. Untreated: untreated cuticle; HCl: cuticle after decalcification (acid treatment); HCl + NaOH: cuticle after deproteinization (acid + alkaline treatment).

TGA measurements confirmed the proximate composition results (Figure 2A,B). In the thermogram of the untreated cuticle, mass loss was observed in three steps. In the two initial weight drops, 3% and 10% of mass loss were attributed to the evaporation of water and polymer degradation, respectively, whereas the third high mass loss, 41%, resulted from the thermal decomposition of calcite (Figure 2A). The final weight loss of the cuticle of *P. pollicipes*, measured at 900 °C, was 52.7%, followed by 75.6% for the cuticle after decalcification and 83.7% for the cuticle after deproteinization. In general, the temperature at which the maximum combustion of organic matter occurred (DTGmax) was between 350 °C and 370 °C, and the thermal decomposition of calcite was 800 °C (Figure 2B). The curve obtained for the untreated cuticle was similar to the ones obtained for decapods [36] and marine isopod cuticles [37], where the separation of the components water, organic matrix and calcium carbonate corresponded to similar weight losses and ranges of temperature. The curves obtained for the treated cuticle showed an initial mass drop of about 10% due to the loss of entrapped water, followed by a reduction of 55% (demineralized cuticle) or 65% (demineralized and deproteinized cuticle) due to the decomposition of organic matter, as proteins and chitin (Figure 2A). These results are compatible with the ones observed for chitin extracted from different crustacean sources (crab, squilla and lobster) [11,38], where mass losses ranged from 8% to 10% in the first stage, from approx. 44% to 70% in the second stage and the maximum degradation temperature (DTGmax) ranged from 351 °C to 359 °C. Thermograms of the treated cuticles also indicated the effect of the decalcification by the absence of the third drop. The elemental composition of the ashes confirmed this result with the removal of the Ca peak (Figure 2C).

**Figure 3:**
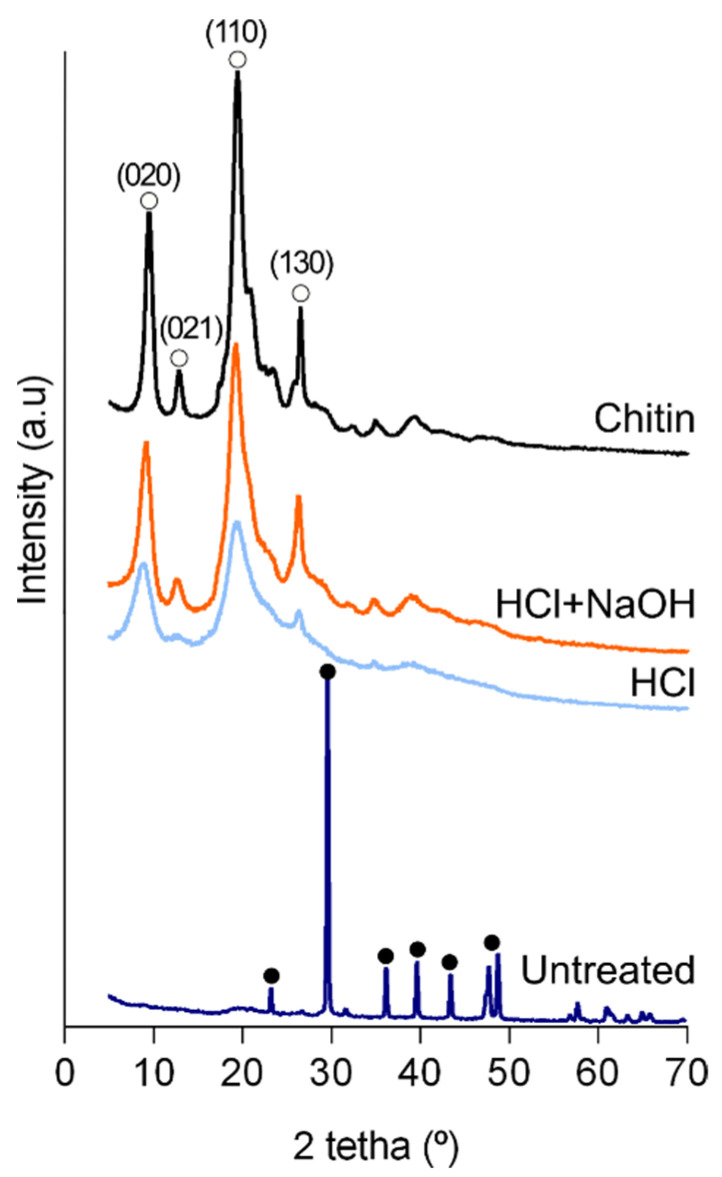
X-ray diffraction patterns of the powdered cuticle. Untreated: untreated cuticle; HCl: cuticle after the decalcification (acid treatment); HCl + NaOH: cuticle after deproteinization (acid + alkaline treatment); Chitin: commercial shrimp chitin. Full circle: peak corresponding to calcite (ICDD # 01-085-1108); Open circle: peak corresponding to ɑ-chitin [39].

X-ray diffractometry spectra revealed that calcite was the main inorganic component as found in the tergum plate (Figure 3 and Figure S2), indicating a similar inorganic composition in the calcified structures of this animal as previously suggested [31]. With the removal of mineral components, two sharp peaks (9° and 19°) and two weak ones (12° and 26°) were observed corresponding to chitin in the ɑ-form [34,39]. These peaks were more pronounced after deproteinization. This result is in accordance with chitin isolated from other crustaceans, anthozoans (i.e., corals) or, considering arthropods’ terrestrial representatives, from insects and spiders, allowing to confirm the effectiveness of the chemical treatments [3,11,40,41].

**Figure 4:**
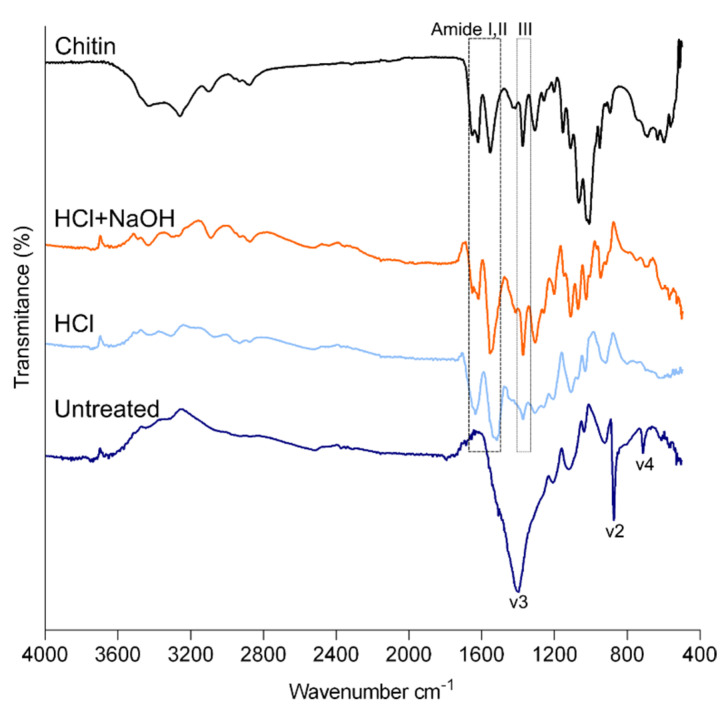
FTIR spectra of powdered cuticle of *P. pollicipes*. Untreated: untreated cuticle; HCl: cuticle after the decalcification (acid treatment); HCl + NaOH: cuticle after deproteinization (acid + alkaline treatment).

FTIR spectroscopy was also performed to investigate the composition of the cuticle. The cuticle IR spectrum resembled the spectra of barnacle plates [42] by exhibiting the following principal calcite peaks: v3 at 1396 cm^−1^, v2 at 871 cm^−1^ and v4 at 711 cm^−1^ (Figure 4). After the two-step treatment, the spectrum was similar to the one acquired for chitin extracted from commercial crustacean species [11,38]. It showed the main signals of chitin: the characteristic split of the amide I band (mainly associated with C=O stretching vibrations) corresponding to the peaks at 1663 cm^−1^ and 1620 cm^−1^ and the signals of amide II and III, at 1556 cm^−1^ and 1373 cm^−1^, respectively. The referred amide I vibration of chitin with a splitting pattern in two components is assigned to chitin in the α–form, in contrast with β which would have an undivided single peak and γ form with an incomplete divided peak [43]. Nevertheless, the other characteristic bands of commercial chitin related to O-H stretching (3496–3372 cm^−1^), N-H stretching (3265–3100 cm^−1^) and CH ring stretching resulting from the deformation of the β-glycosidic bond (890–895 cm^−1^) [44] were less clear or absent (Figure 4). X-ray data also showed that the intensive peak of commercial chitin was broader than the cuticle after the two treatments. Both analyses may indicate that chitin purity after the treatments was lower than the commercial one.

### 2.3. Organization and General Structure of the Cuticle

**Figure 5:**
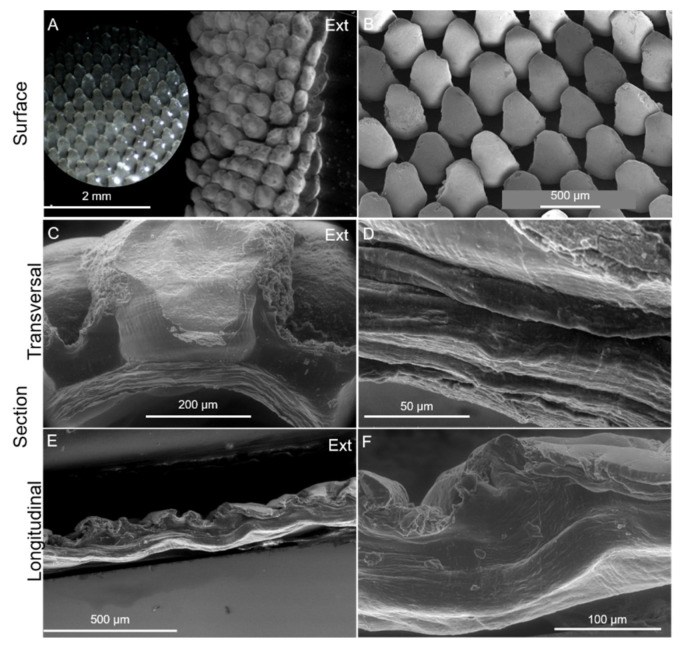
Imaging of the cuticle of *P. pollicipes*. (**A**): ESEM of the external surface of the cuticle in its natural state and a detail of the surface (optical microscopy image); (**B**): SEM of the surface of the cuticle cut longitudinally and spread open showing the semi-vertical/diagonal orientation of the scales. (**C**,**D**): ESEM of a cross-transversal section exposing the insertion of a scale into the organic layer and a detail of the laminar structure, respectively; (**E**,**F**): ESEM of the cross-longitudinal section exposing circumferential ridges. Ext: external surface which is in contact with the environment.

Imaging mineralized biological materials is a challenging process due to the complex interaction of organic and inorganic components forming their architecture. This work showed that the cuticle structure can be captured and interpreted using a combination of imaging analysis and histology, thereby disentangling the artefacts related to sample preservation and preparation for imaging. Macro-photo images displayed in Figure 10 illustrate the tubular shape of the cuticle covering the elongated stalk, which can measure up to 65 mm in length and 11 mm in diameter [31,45]. SEM micrographs of a transversal section showed that it consists of an inner cuticular zone composed of homogeneous compacted layers, measuring approximately 100 µm in thickness (Figure 5C,D), and an outer cuticular zone measuring around 400 µm which surrounds numerous scales (Figure 5C). The scales are arranged in parallel and oriented semi-vertically and cover the entire outer surface of the cuticle, even so presenting small differences (Figure 5A,B). At the animal base, the scales are smaller and the space between them is greater owing to stretching and contracting [31] (Supplementary Material, Figure S3). The longitudinal section displayed a series of circumferential ridges possibly related to the tubular form of the cuticle (Figure 5E,F). The elemental distribution was analyzed in the two zones by energy-dispersive X-ray spectroscopy. The spectra showed strong signals from Ca, C, Si and O and small amounts of S, Al, Fe, Na and Mg. The Ca signal was higher in the scales’ region, whereas Si signals were higher in the outer cuticular zone (Supplementary Material, Figure S4).

**Figure 6:**
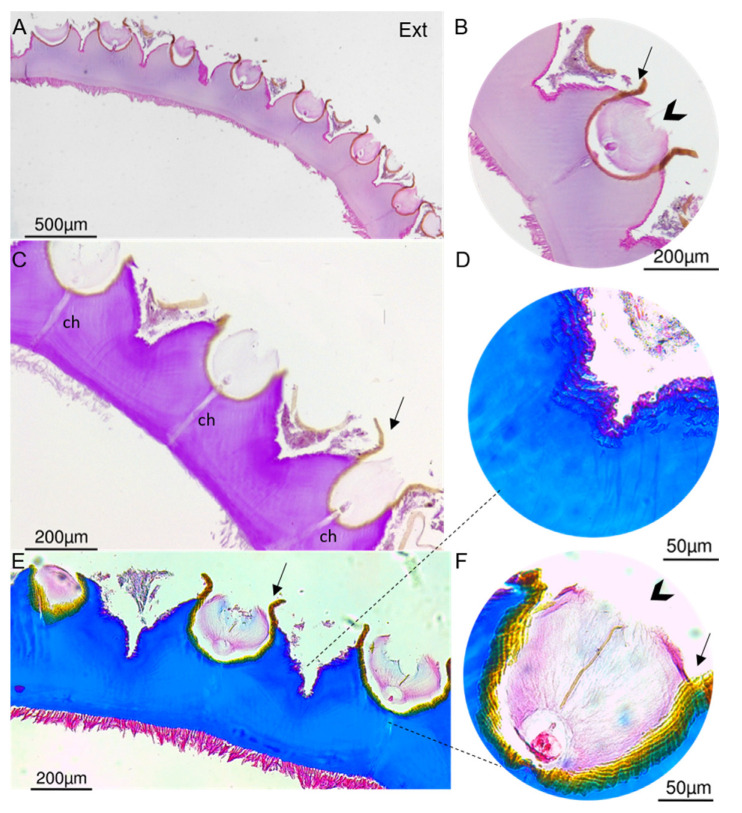
Histological cross-transversal section of the decalcified cuticle of *P. pollicipes* on paraffin section. (**A**,**B**). Section stained with H&E showing the base of the elevations where the scales are inserted and a “film” (arrow). (**C**). Section stained with periodic acid–Schiff exhibiting channels (ch) crossing the cuticle. (**D**–**F**). Cross-section stained with Masson’s trichrome showing the brown sheath (arrowhead) and protein content between the insertion of the scales (**F**). Ext: external surface which is in contact with the environment.

Histology, carried out to complement the analysis of the cuticle structure, revealed an acellular structure (Figure 6). Images of a transversal section of the decalcified cuticle confirmed that the scales are inserted in semi-globular depressions (Figure 6) embedded in a brown sheath (Figure 6B,C,F), and possibly involved by a thin “film” which remained after scale removal. This was also shown in resin sections where only the bases of the scales were stained blue and magenta with Giemsa and Periodic acid–Schiff (PAS), respectively, and the top remained unstained (Supplementary Material, Figure S5A,B). The channels observed crossing the cuticle from the epidermis to the base of the scale may be involved in the secretion of materials for cuticle development. Concerning cuticle composition, staining with H&E suggests that cuticle contains basic (acidophilic) binding sites, commonly stained pink by eosin [46], and suggests that cuticle contains acidic proteins between scales, commonly stained pink by Masson’s Trichrome Staining (Figure 6B,D), whereas the bulk cuticle contains chitin and glycoproteins (magenta with PAS) (Figure 6C) and collagen fibers (blue with Masson’s trichrome) (Figure 6E). A previous analysis of *P. polymerus* integument suggested a similar composition, i.e., chitin, reinforced with elastin or collagen-based proteins to withstand tension [47].

### 2.4. Fine Structure of the Cuticle

**Figure 7:**
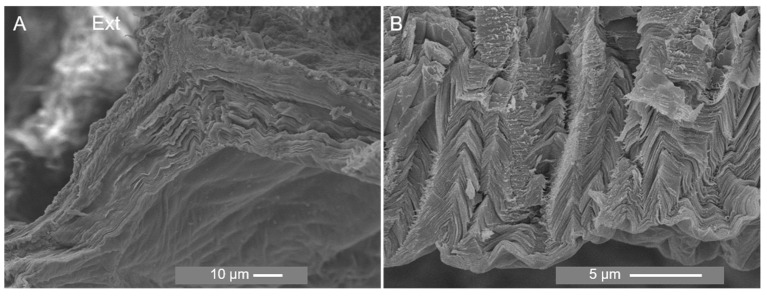
SEM analysis of the section of the cuticle of *P. pollicipes* at magnification of ×1000 (**A**) and ×5000 (**B**). Ext: external surface which is in contact with the environment.

SEM imaging exhibited a wrinkled and rough appearance in the inner and outer cuticle surfaces. Although the cuticle appeared to be better preserved in the ESEM mode, conventional electron microscopy imaging revealed an endocuticular zone constituted of successive layers parallel to the surface (Figure 7A) and possibly microfibrils involved in sheet connections (Figure 7B). It also showed a compressed cuticle and a “zig-zag” pattern not observed in the environmental mode which could be attributed to sample dehydration. This pattern is also manifested in histological sections (Supplementary Material, Figure S5C). Similarly, by comparing paraffin and resin sections, the distance between scales decreased in the former, and, therefore, preparation with paraffin changed the structure of the cuticle (Supplementary Material, Figure S5). Moreover, dehydration led to a less preserved outer cuticular zone, as exhibited in several images of the cross-section of the cuticle where it is observed a “disintegration” of this zone (Supplementary Material, Figure S6).

Despite the SEM and ESEM mode differences, overall, the multi-layered laminar structure is similar to hard and soft chitin-based marine materials originating from several marine phyla [48,49,50].

**Figure 8:**
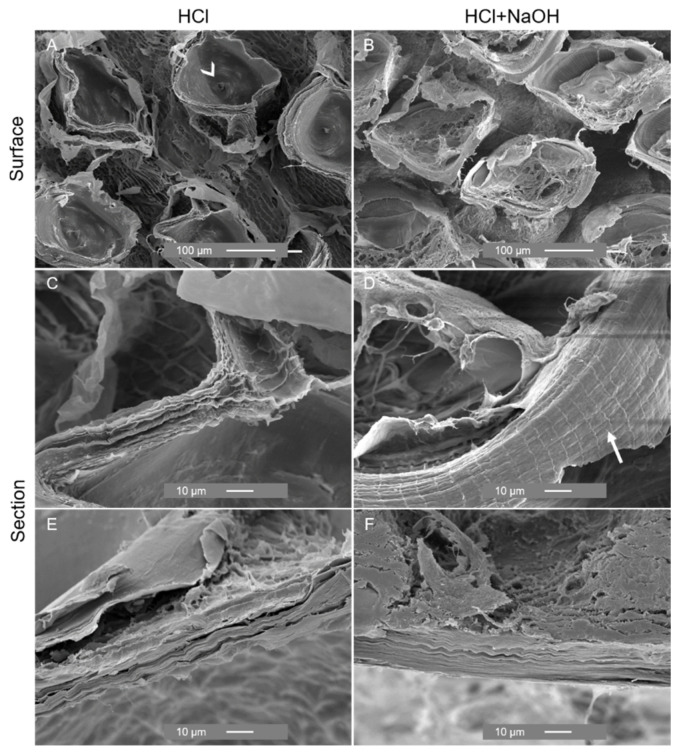
SEM analysis of the external surface and section of the cuticle of *P. pollicipes* after decalcification (acid treatment) ((**A**): surface, (**C**,**E)**: cross-section) and deproteinization (acid + alkaline treatment) ((**B**): surface; (**D**,**F**): cross-section).

With the decalcification and removal of the scales, SEM images showed significant changes in the outer zone of the cuticle, exposing rounded holes with an opening at the base possibly related to the presence of channels for the delivery of material for scale development (Figure 8A) and exhibited fine sheets (Figure 8C). Deproteinization resulted in the shrinkage of the cuticle, as shown by the decreased distance between the holes (Figure 8B), and it evidenced the organized laminar structure (Figure 8F) and a lattice pattern of the sheet (Figure 8D). Similar changes in morphology were observed during the process of chitin extraction from the exoskeleton of the shrimp *Litopenaeus vannamei*. The exoskeleton of this shrimp exhibited a heterogeneous morphology characterized by white spots composed of calcium. After demineralization and deproteinization treatments, the white spots were replaced by rounded holes, and the fibrous nature of the material was revealed, being in accordance with the observation of a previous study [51].

### 2.5. Mechanical Properties

**Figure 9:**
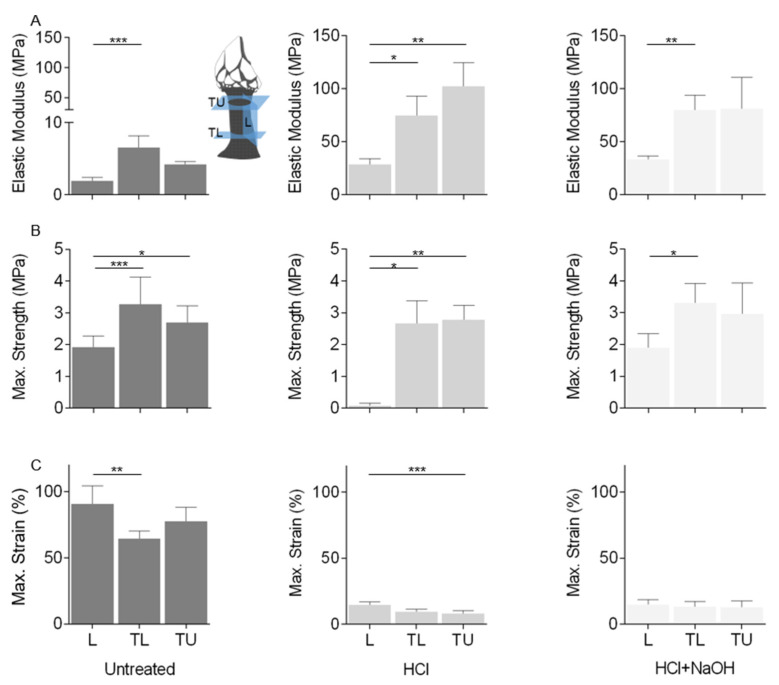
Tensile properties of the cuticle: (**A**) elastic modulus, (**B**) strength at maximum load and (**C**) strain at maximum load. Tests carried out at three regions: longitudinal plane (L), transversal plane at low-body level (TL) and transversal plane at upper-body level (TU), and three conditions: Untreated: untreated cuticle (Untreated); cuticle after the decalcification (acid treatment, HCl); cuticle after deproteinization (acid + alkaline treatment, HCl + NaOH). Data presented as mean ± standard deviation. *: *p* < 0.05; **: *p* < 0.01; ***: *p* < 0.001.

Uniaxial tensile tests were conducted at the longitudinal plane and the transversal plane at the low-body level and at the upper-body level of the cuticle to access its mechanical properties (Figure 9). The elastic modulus of the untreated cuticle varied between 1.91 and 6.55 MPa, and the maximum tensile strength was between 1.92 and 3.28 MPa. Significant differences were found between the longitudinal and the transversal lower directions, with the former showing the lowest values. The tensile strain at maximum load ranged from 64.42 to 90.75%, indicating that the cuticle can sustain high deformation before fracturing (ductile behavior), particularly in the longitudinal direction with an elongation of approx. 100%.

The decalcification process led to a decrease in cuticle thickness and significant differences in the three properties and directions. An increase in the material stiffness of 10 times higher (28.71 to 102.26 MPa) was observed, particularly in the transversal directions, leading to a more brittle fracture behavior (strain at maximum load: 7.83 to 14.77%). These results indicate that this treatment led to the removal of calcified components and changes in protein composition related to the removal of collagen or changes in protein properties by increasing the cross-linking of the cuticle proteins, possibly manifested in the outer and inner zones of the cuticle. Despite the chemical and structural changes observed with the deproteinization, it appeared that this treatment had a lesser effect on the mechanical properties, which remained, in general, similar to the decalcification (elastic modulus: 33.55 to 81.05 MPa, max. strength: 1.89 to 3.31 MPa; strain at maximum load: 12.86 to 14.92%), indicating that the alkaline treatment had a smaller effect when compared with the acid treatment.

Comparing these results with other marine materials is limited due to the lack of studies on their mechanical properties. For instance, the elastic modulus of *P. pollicipes* cuticle is in the range of values described for the soft cuticle formed during *C. sapidus* exoskeleton replacement, which corresponds to the initial phase of new cuticle formation without the initiation of the mineralization process [52]. These values are also comparable to resilin, an elastomeric protein found in many insect cuticles and other arthropods. This protein has several functions such as the generation of deformability and flexibility in membrane and joint systems, the storage of elastic energy in jumping and catapulting systems and the sealing of wounds in a traumatic reproductive system. It is also present in many compound eye lenses and, therefore, is suggested to be a very suitable material for optical elements because of its transparency and amorphousness [53]. After decalcification, the elastic modulus approximates to the byssal mussel thread, a biopolymer that adheres to hard surfaces with high strength and extensibility, allowing mussels to withstand the large and repetitive force produced by waves [52,54], inspiring scientists towards the development of biomimetic materials and adhesives, including for biomedicine applications [55].

Overall, the results of the mechanical properties indicate that despite the predominantly inorganic composition of the cuticle (>70%), it presented low rigidity, meaning that polymers determine most of their mechanical properties. Significant differences between the longitudinal and transversal directions suggest variability in material properties in different cuticle directions (anisotropy). Previous works point out that a new cuticle is formed in the upper section of the stalk, whereas there is high contraction and scratching in the lower section manifested by the low scale size and the short distance between them [31,56]. Additionally, the lower region of the cuticle is closer to the animal base that attaches to the substrate. Therefore, material properties in this region can be affected by adhesion [57]. The characteristics in the lower part of the animal may explain the high elastic modulus, max. strength and lower strain at maximum load in the transversal lower direction. Within the longitudinal direction, it is also possible that fibers running in this direction may present distinct properties related to the ability of the peduncle to serve as a pressure-containing vessel [47]. It would also be interesting to analyze the internal surface of the cuticle as previous works described differences between cuticle topographies, which were correlated with different abilities for cell adhesion and proliferation [19].

### 2.6. Flexible Cuticles as Biomimetic Models for Tissue Engineering and Regenerative Medicine Applications

Materials of crustacean origin are composites comprising multiple constituents with different physical and chemical properties tuned to meet various functionality requirements [10]. Their study has been centered on their rigid exoskeletons and recently on the replacement exoskeletons formed during growth, in which their mechanical and structural properties must suit exoskeleton requirements (i.e., rigid or hydrostatic) [52]. For example, sustainable chitin sources such as the “soft-skeletons” of commercial crabs or the tube-like porous chitin structures of spiders, produced during molting, are currently being evaluated as naturally prefabricated materials for biomedical applications with minimum processing [19,58]. In *P. pollicipes*, chemical treatments can tune cuticle mechanical properties to fulfil the requirements for chitin-based tissue engineering approaches. Nevertheless, the sustainability of this resource should be analyzed as harvesting is not ecologically sustainable [59]. Attempts have been made in the last decade to cultivate *P. pollicipes* in captivity but this remains challenging [60].

Considering biomimetic approaches, the elastic arthrodial membranes that connect the rigid cuticles in the American lobster *Homarus americanus* which provide agility in locomotion are designed to act as soft yet strong and tough materials for a variety of biomimetic applications, such as soft body armor with maximized mobility, soft robotics and artificial tissues, among others [12]. The above examples illustrate the potential of marine crustacean functional diversity to provide a foundation for biomimetic approaches to develop optimized bio-inspired materials. The cuticle of *P. pollicipes*, described as a predominantly inorganic, flexible and tubular-shape material, may motivate the design of synthetic flexible materials for biomedical applications such as tissue engineering approaches for bone, vascular or skin tissues. Other marine examples could also be studied, such as tubes built by polychaete worms. They are generally composed of layers containing exogenous materials, fibers and glue materials forming plywood structures that support and protect their residents of diverse mechanical settings such as high-energy wave environments or sediment mobility in the case of burrowing species [48,49]. In another example, the hierarchical structure of the organic tunic of the ascidian *Halocynthia roretzi*, a composite comprising a thin sclerotized cuticular layer and a cellulose-based layer has also been proposed as an interesting biomaterial [61]. Therefore, the marine environment is a source of inspiration, with a particular focus on marine invertebrates that account for huge marine diversity [62].

## 3. Material and Methods

### 3.1. Sample Collection and Preparation

**Figure 10:**
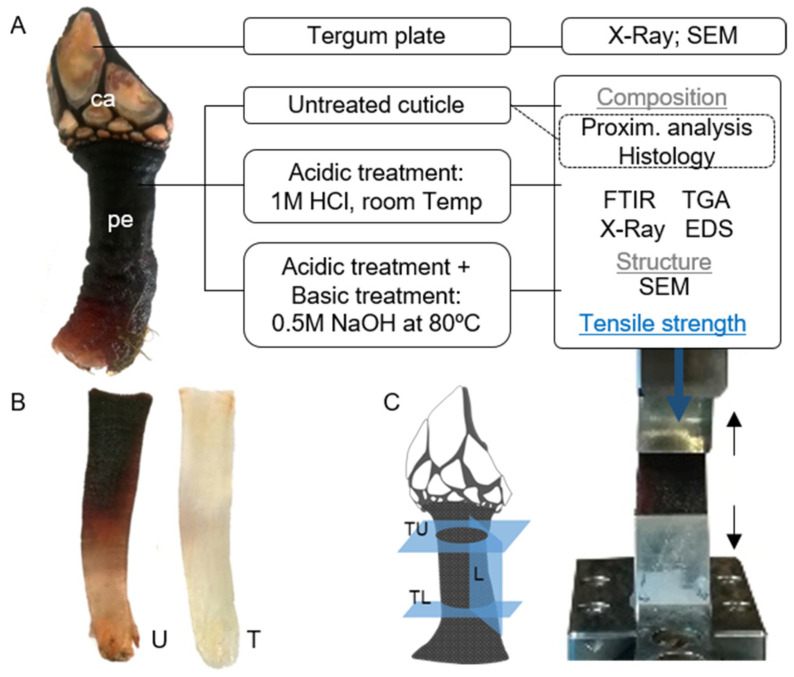
(**A**). Goose barnacle *Pollicipes pollicipes* (all animal: ca: capitulum; pe: peduncle or stalk) and schematic representation of the analyses carried out for the characterization of the integument of *P. pollicipes* (tergum plate and cuticle); (**B**). illustration of the cuticle after the treatments (U: untreated cuticle; T: cuticle after the acid and alkaline treatments); (**C**). Illustration showing how the cuticle was cut for tensile testing (L: cuticle in the longitudinal direction; TL: cuticle in the transversal lower direction; TU: cuticle in the transversal upper direction) and an image of a specimen in the grips under tensile load.

*P. pollicipes* was collected in a rocky intertidal zone of the NW coast of Portugal (location “Praia da Agudela”; 41°14’19.4″ N, 8°43’33.3″ W), transported to the laboratory in sterilized plastic bags and then sorted to select adult individuals (adult size: rostro-carinal length > 15 mm) [23]. The plates covering the capitulum (Figure 10) were separated from the peduncle, and the soft tissues were removed from both parts. The tergum plate and cuticle were rinsed repeatedly with abundant distilled water, and the debris and byssal threads from mussels were removed from the cuticle. The cuticle pieces were either kept hydrated in a saline solution or fixed by immersion in 4% paraformaldehyde until imaging analysis. They were also preserved at −20 °C for later processing, namely, for the removal of minerals and proteins, preparation for chemical analysis and other imaging techniques. In this regard, cuticle pieces were firstly processed with an acid treatment (demineralization) of 1 M HCl solution at a ratio of 1:10 (*w*/*v*), at room temperature for 4 h, followed by several washes in distilled water until pH neutrality. Samples were inspected under a light microscope, and scales still attached to the cuticle were carefully removed with forceps. Next, a group of samples submitted to the previous treatment was processed with an alkaline treatment (deproteinization) of 0.5 M NaOH solution at a ratio of 1:20 (*w*/*v*), at 80 °C, for 2 h, followed by several large-volume washes in dH20 until pH neutrality (Figure 10). For some imaging techniques, the tergum plate and untreated and treated cuticles were air-dried at 37 °C for 72 h or freeze-dried. For chemical examination, air-dried samples were prepared by crushing and grinding them into a fine powder.

### 3.2. Plates Characterization

The outer surface and cross-section of a dried fragment of the plate were sputter-coated with platinum using an EM Leica ACE600 Sputter coater and examined in a scanning electron microscope (SEM) JSM-6010 LV (JEOL, Tokyo, Japan), with an acceleration voltage of 10 kV, at different magnifications. The relative chemical elements of the outer surface and cross-section of the plate were measured using an energy-dispersive spectroscope (EDS) (INCAx-Act, PentaFET Precision, Oxford Instruments, Tokyo, Japan) at an energy of 10 keV coupled with SEM. To analyze the crystalline phases, diffraction measurements of the powders were performed using a conventional Bragg–Brentano X-Ray diffractometer (Bruker D8 Advance DaVinci, Karlsruhe, Germany) equipped with Cu Kα radiation. Data sets were recorded in the 2θ range of 10–80° with a step size of 0.02° and 1 s for each step.

### 3.3. Cuticle Characterization

#### 3.3.1. Proximate Composition and Estimation of Chitin Content

To determine the proximate composition, untreated cuticle was freeze-dried, ground and homogenized before the analysis. All chemical analyses followed the Association of Official Analytical Chemists methods [63] and were performed in duplicate. The samples were analyzed for dry matter (105 °C for 24 h), ash by combustion in a muffle furnace (Nabertherm L9/11/B170, Bremen, Germany; 550 °C for 6 h), crude protein (N × 6.25) using a Leco nitrogen analyzer (Model FP-528, Leco Corporation, St. Joseph, MI, USA) and lipid content by petroleum ether extraction using a Soxtherm Multistat/SX PC (Gerhardt, Germany). The carbohydrate content was estimated based on the remaining fraction: Carbohydrate = 100% − (% protein + % fat + % ash + % Moisture). The chitin content was estimated based on the ratio between the weight of the dried cuticle after the abovementioned demineralization and deproteinization treatments and the dry weight of the untreated cuticle.

#### 3.3.2. Thermal Gravimetric Analysis (TGA) and EDS

Powders of untreated and treated cuticle were submitted to TGA using a STA7200 Simultaneous Thermal Analyzer (Hitachi, Japan). The analyses were performed at a heating rate of 10 °C min^−1^ from 50 °C to 900 °C in an air atmosphere and were performed in triplicate. The ash was used for EDS analysis for mineral determination, following the procedure described previously (Section 3.2).

#### 3.3.3. Attenuated Total Reflection–Fourier Transform Infrared (ATR-FTIR) Spectroscopy

Untreated and treated cuticle powders were analyzed by ATR-FTIR. The infrared spectra were obtained with a Shimadzu-IR Prestige 21 spectrometer (Kyoto, Japan), with an attachment of total attenuated internal reflection, in the spectral region of 4000–500 cm^−1^ using a resolution of 4 cm^−1^, with each spectrum being the average of 32 scans. The samples were analyzed in the transmission mode.

#### 3.3.4. X-Ray Diffraction (XRD)

Following the procedure described previously (Section 3.2), untreated and treated cuticle powders were scanned from a 2θ range between 5° and 70° with a step size of 0.02° and 1 s for each step.

#### 3.3.5. Histology

For histology, cuticle located at the mid-body level was analyzed. Briefly, 4% paraformaldehyde-fixed cuticle was immersed in 1 M HCl for 72 h for demineralization followed by dehydration in a sequence of graded ethanol, embedding in paraffin wax and sectioning at 10 µm. Non-demineralized cuticle was also prepared by fixation for 7 days in 3.7% paraformaldehyde, followed by dehydration with ethanol, embedding in methacrylate resin and sectioning at 30 µm. Cross-transversal paraffin sections were stained with Hematoxylin-eosin (H&E) for cellular detection, periodic acid–Schiff (PAS) for carbohydrate detection and Masson’s trichrome for collagen detection. At the same time, cross-transversal resin sections were stained with Giemsa for cellular detection. These stains were used to identify putative different cuticular layers described in arthropod cuticles [46,64,65]. All sections were examined and photographed using a Leica DM750 microscope (Wien, Austria).

#### 3.3.6. Environmental Scanning Electron Microscopy (ESEM) and Scanning Electron Microscopy (SEM)

ESEM micrographs were obtained using a Quanta 650 FEG Environmental SEM with EDXS analysis (Fei Company, Hillsboro, OR, USA) under service acquisition from the International Nanotechnology Laboratory (INL, Braga, Portugal). Before examination, hydrated cuticle located at the mid-body level was sectioned to expose the internal component (cross-section). Then, the cuticle was imaged, namely, the external surface in the longitudinal direction and the exposed cross-section in the longitudinal and transversal directions (Figure 10C). Samples were placed into a drop of water on the Peltier cooling stage at 4 °C, 100 to 760 Pa of water vapor and an acceleration voltage of 10 kV at different magnifications. Chemical analysis was measured on the outer surface and cross-sections at an energy of 10 keV. For SEM (JSM-6010 LV, JEOL, Kyoto, Japan), dried untreated and treated cuticles were sectioned as indicated above, and the external surfaces and the exposed cross-sections were sputter-coated with an electrically conducting layer of gold using the EM Leica ACE600 Sputter coater (Wein, Austria). The cross-section imaging results are presented only for the transversal direction, as the results for the longitudinal direction were similar or less clear.

#### 3.3.7. Mechanical Testing

The mechanical properties of the untreated and treated cuticle were determined by performing tensile tests using an Instron 5543 universal mechanical testing machine equipped with a pneumatic BioPlus tensile grips system and were performed in a minimum of six samples per condition. Rectangular 15 mm × 12 mm specimens were cut in the longitudinal and transversal directions with a lamina (Figure 10C) and left in 20% ethanol until analysis. Their thickness ranged from 250 μm to 600 μm, measured by a digital caliper. A 10 mm distance between grips was used, and the tensile modulus, ultimate tensile strength and strain at maximum load of the hydrated cuticle pieces were measured using a 1 kN load cell at a crosshead speed of 2 mm min^−1^.

#### 3.3.8. Statistical Analysis

For proximate composition and tensile testing, data are presented as the mean ± standard deviation. For tensile testing, non-parametric Kruskal–Wallis tests, following Dunn’s multiple comparisons, were used to determine the significance level with a *p*-value less than 0.05 (*p* < 0.05) being considered statistically significant. Statistical analyses were performed using GraphPad Prism 8.0.1 software.

## 4. Conclusions

Based on the combination of compositional, structural and mechanical studies of the cuticle of *P. pollicipes*, detailed knowledge of this marine invertebrate material was obtained. Cuticle composition is similar to well-characterized rigid cuticles from harvested crustacean mobile species. Structurally, it is a hybrid flexible material, predominantly inorganic, possibly in the form of scales organized to fully cover the polymeric part. This arrangement may provide abrasion resistance to the effects of sand and debris caused by crashing waves, protection against predation and the protection of larvae that settle on the surface of the animal. The polymeric component relying on chitin, collagen and elastin-based structural proteins, organized in a laminar structure, may provide the properties necessary to deal with the crashing of waves and support the body by maintaining its shape. Further studies could elucidate the biomimetic potential of the cuticle as a predominantly inorganic material with elastic properties aiming to be explored for the development of high mechanical performance textiles or biomaterials for tissue engineering applications, among others.

## Figures and Tables

**Table 1:** Proximate composition of the untreated cuticle expressed in percentage (%) of dry weight. Data presented as mean ± standard deviation.

% Dry Weight			
Ash	Protein	Lipids	Carbohydrates
75.16 ± 0.86	19.91 ± 0.13	0.33 ± 0.01	3.51 ± 0.86

## Supplementary Materials

The following supporting information can be downloaded at: https://www.mdpi.com/article/10.3390/md21020096/s1, Figure S1. Imaging of the cuticle of P. pollicipes; Figure S2. X-ray powder diffraction of the plate of *P. pollicipes*; Figure S3. Aspect of the outer surface of the cuticle of *P. pollicipes*; Figure S4. Elemental composition weight (%) of the cross-section and surface of the untreated cuticle of *P. pollicipes*. Figure S5. Histological section of the cuticle of *P. pollicipes.* Figure S6. SEM analysis of the cross-section of a transversal and longitudinal cut of the cuticle of *P. pollicipes* at different magnifications.
