# Supplementary material for: Structure and Composition of the Cuticle of the Goose Barnacle Pollicipes pollicipes: A Flexible Composite Biomaterial
Source: Mar Drugs. 2023 Jan 29;21(2):96. doi: 10.3390/md21020096 (PMC9968147; doi:10.3390/md21020096)
Supplement: Supplementary file 1 [file marinedrugs-21-00096-s001.zip › marinedrugs-2021645-supplementary.pdf]

marine drugs

Structure and Composition of the Cuticle of the Goose Barnacle *Pollicipes pollicipes*: A Flexible Composite Biomaterial

Mariana Almeida [1,2,*], Emanuel M. Fernandes [1,2], Catarina F. Marques [1,2], Flávia C. M. Lobo [1,2], Rita O. Sousa [1,2], Rui L. Reis [1,2] and Tiago H. Silva [1,2,*]

1 3B's Research Group, I3Bs–Research Institute on Biomaterials, Biodegradables and Biomimetics, University of Minho, Headquarters of the European Institute of Excellence on Tissue Engineering and Regenerative Medicine, AvePark, Parque de Ciência e Tecnologia, Zona Industrial da Gandra, Barco, Guimarães 4805-017 , Portugal
2 ICVS/3B's–PT Government Associate Laboratory, 4806-909 Braga/Guimarães, Portugal
* Correspondence: mariana.almeida@i3bs.uminho.pt (M.A.); tiago.silva@i3bs.uminho.pt (T.H.S.)

Supplementary material

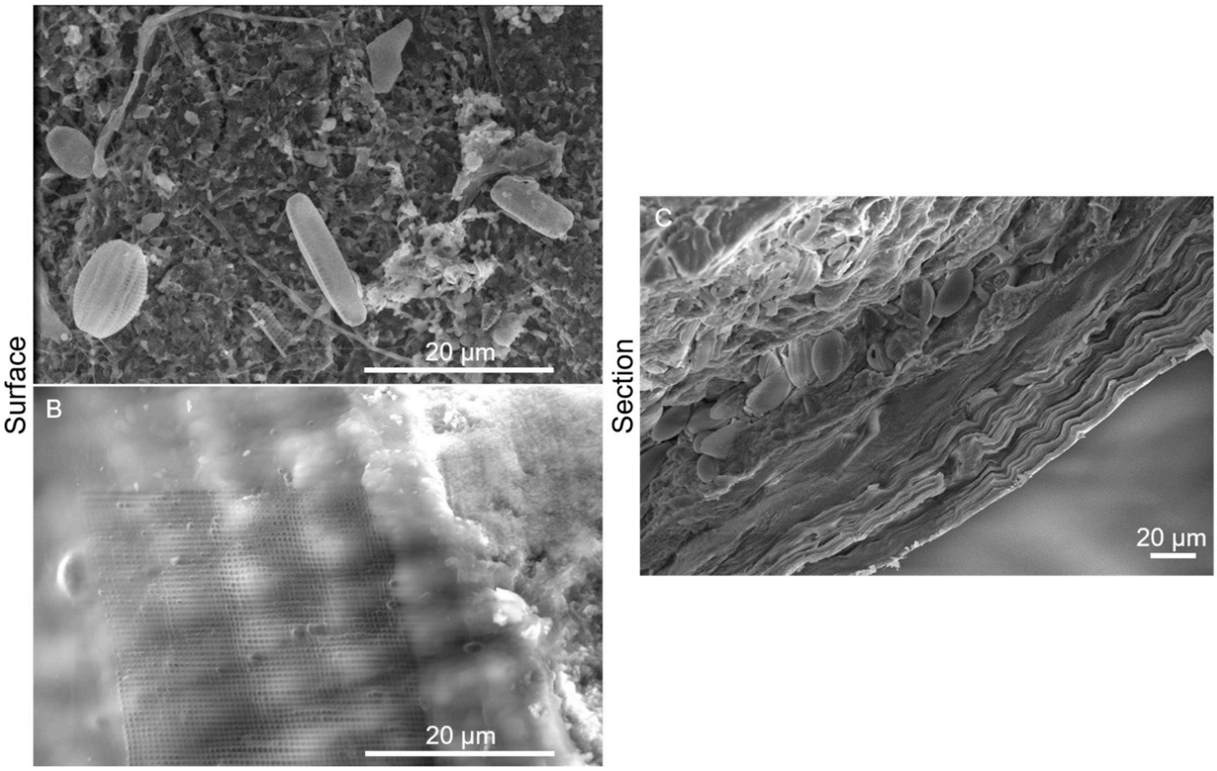

Figure S1. Imaging of the cuticle of *P. pollicipes*. A and B: ESEM of the surface of the cuticle showing the establishment of diatoms (A) and bacteria (B); C: SEM of the cross-transversal section exposing the outer layer of the cuticle with diatoms.

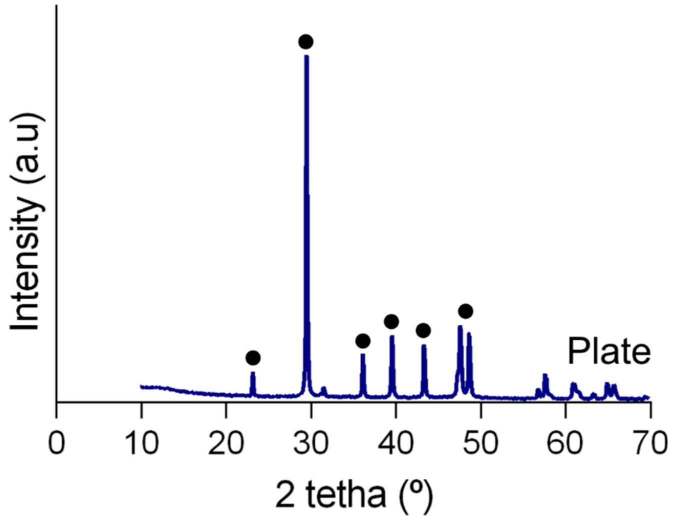

Figure S2. X-ray powder diffraction of the plate of *P. pollicipes*. Full circle: peak corresponding to calcite (ICDD #01-085-1108).

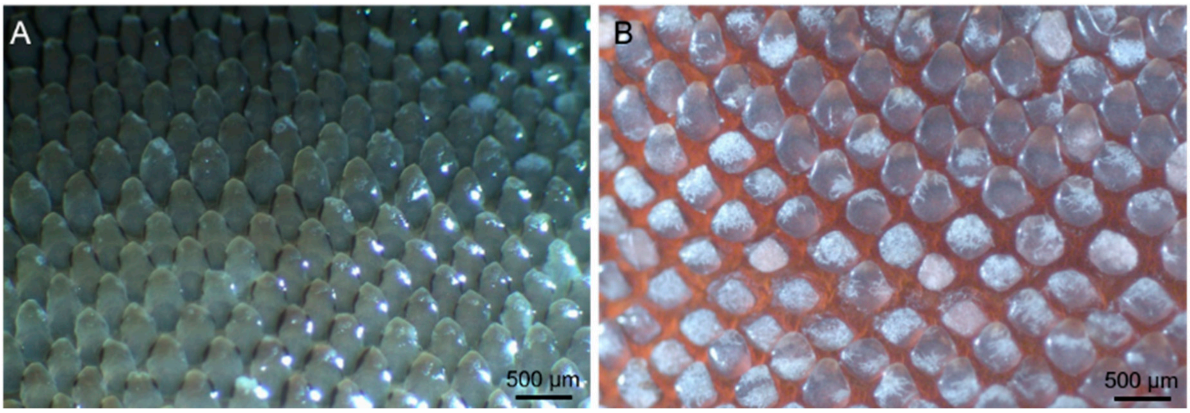

Figure S3. Aspect of the outer surface of the cuticle of *P. pollicipes.* Up-body level (A); low-body level (B).

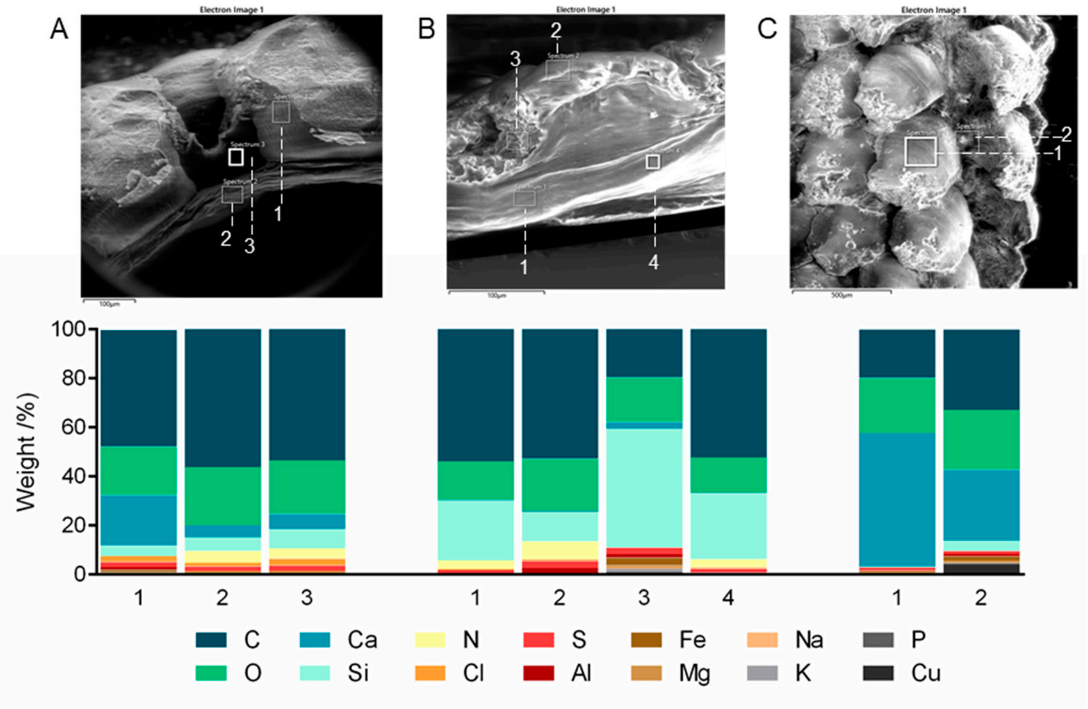

Figure S4. Elemental composition weight (%) of the cross section (A, B) and surface (C) of the untreated cuticle of *P. pollicipes*.

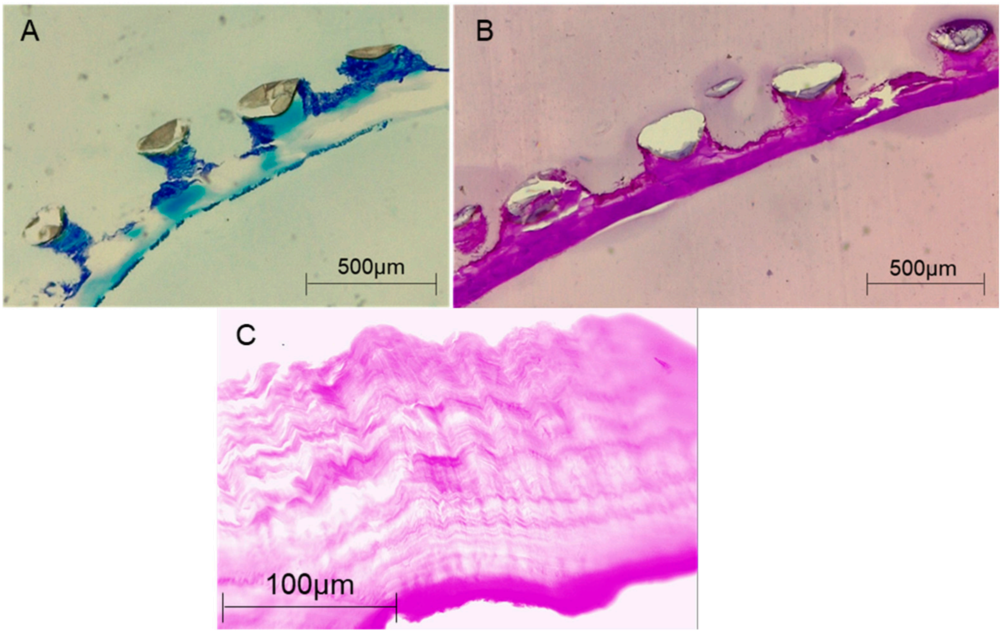

Figure S5. Histological section of the cuticle of *P. pollicipes.* A and B: Sections on resin stained with Giemsa (A) and PAS (B) showing a higher distance between scales. C: Section on paraffin stained with PAS (C) showing the pattern of the layers.

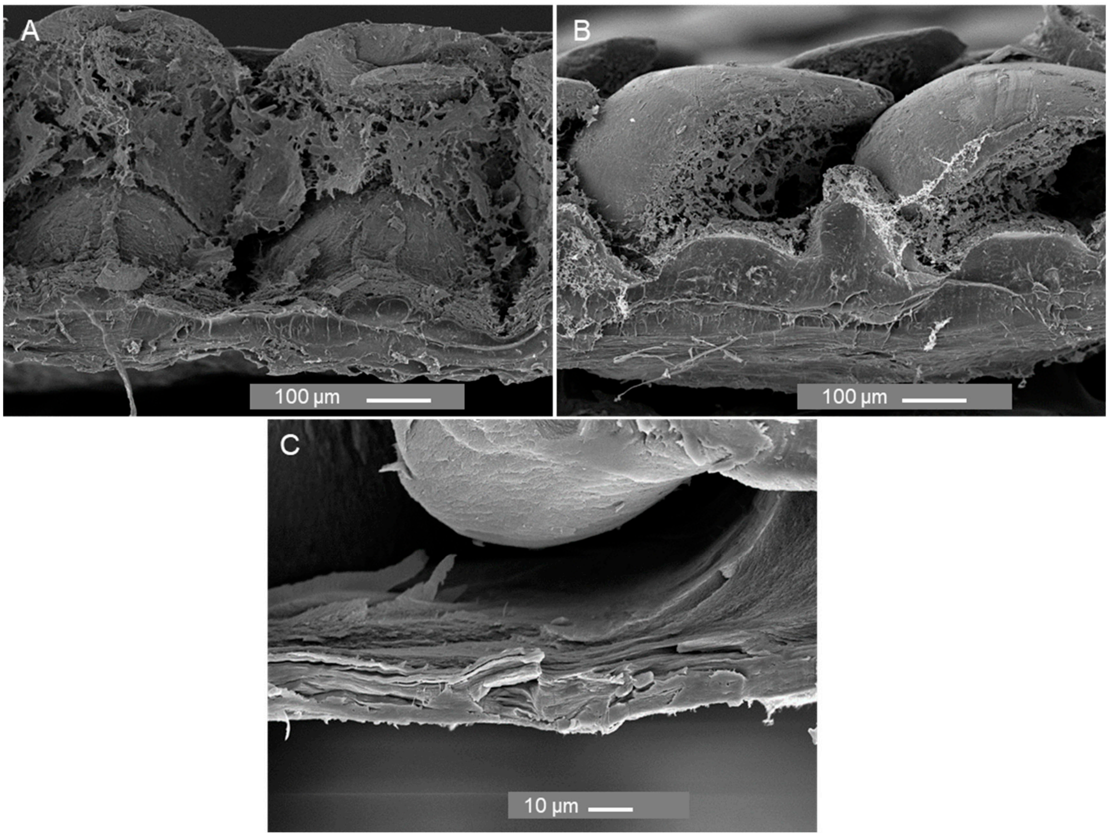

Figure S6. SEM analysis of the cross-section of a transversal (A) and longitudinal cut (B, and C) of the cuticle of *P. pollicipes* at different magnifications. A and B: x150; C: x1000. Note: Dehydration of the samples for SEM observation mode showed the disintegration of the outer cuticular zone which involved the scales as observed in ESEM mode.
